# The predictive value of albumin-based nutritional indices for intestinal ischemia in strangulated abdominal wall hernias: an insight into the novel CALLY Index

**DOI:** 10.1007/s10029-025-03365-0

**Published:** 2025-05-20

**Authors:** Yavuz Selim Angın, Ahmet Murat Şendil, Akile Zengin, Cengiz Ceylan, Mehmet Kılıç, Murat Ulaş

**Affiliations:** 1 Department of General Surgery, Eskişehir Osmangazi University, Eskisehir, Türkiye; 2 Department of Gastrointestinal Surgery, Eskişehir Yunus Emre Hospital, Eskişehir, Türkiye

**Keywords:** Intestinal ischemia, Strangulated hernia, Nutritional indices, CALLY index, Modified glasgow prognostic score, Surgical risk assessment

## Abstract

**Purpose:**

Intestinal ischemia in strangulated abdominal wall hernias is a life-threatening condition that requires early detection and timely intervention. Since both systemic inflammation and nutritional status play a crucial role in surgical outcomes, identifying reliable predictive markers is essential. This study explores the potential of albumin-based nutritional indices—the CALLY Index, Prognostic Nutritional Index (PNI), and modified Glasgow Prognostic Score (mGPS)—to assess their ability to predict the risk of intestinal ischemia and the need for surgical resection.

**Methods:**

We retrospectively analyzed 125 patients who underwent surgery for incarcerated abdominal hernias between January 2015 and August 2024. Patients were categorized based on whether they had intestinal or omental ischemia. Key demographic data, perioperative findings, and laboratory parameters—including CRP, albumin, lymphocyte count, PNI, CALLY, and mGPS—were recorded. We performed univariate and multivariate regression analyses to identify independent risk factors, while ROC analysis was used to determine the optimal cutoff value for the CALLY Index.

**Results:**

Out of 125 patients, 66 (52.8%) were female, and the median age was 67 (58–76) years. Intestinal ischemia was found in 73 (58.4%) of the patients, while 23 (18.4%) had only omental necrosis. In the multivariate regression analysis, GPS 2 (OR: 19.299, *p* = 0.015) and CALLY < 2.5 (OR: 5.397, *p* = 0.017) were identified as independent predictors of intestinal ischemia. ROC analysis showed that a CALLY value below 2.5 had strong predictive power (AUC: 0.828, 95% CI: 0.753–0.902, *p* < 0.001).

**Conclusion:**

Our findings suggest that the CALLY Index and mGPS are valuable tools for predicting intestinal ischemia in strangulated abdominal hernias. By incorporating inflammatory and nutritional markers into risk assessment, these indices can assist in early diagnosis and timely surgical decision-making.

## Introduction

Inflammation and nutritional state are both influential factors when it comes to determining the prognosis of surgical patients [1]. Systemic changes arising from acute inflammation may as well affect the immune response and have an impact on the severity of tissue damage and recovery processes [2].

C-reactive protein (CRP) is a positive acute-phase reactant stimulated by proinflammatory cytokines (IL-6, TNF-α, IL-1β) and is therefore a valuable indicator of inflammation [3].

In contrast, serum albumin is regarded as a negative acute-phase reactant that decreases during inflammation and hence reflects a substantial biochemical parameter of the patient's nutritional status [4].

Whereas the CRP levels shoot up, serum albumin levels drop during acute inflammation marking their significance in guiding an appraisal of the severity of inflammation and the general clinical state of a patient.

Another condition frequently observed according to inflammatory response is lymphopenia [5]. A notable decrease in lymphocytes count is visible during acute inflammation. This condition may occur due to the entry of lymphocytes into the margination and redistribution process, the impact of the proinflammatory cytokines on lymphocyte apoptosis, and a boost in the plasma cortisol because of inflammation [6]. Lymphopenia is overtly considered a poor prognosis indicator, notably in sepsis and severe infections, and seems to pose an important biomarker for the determination of disease severity in surgical patients [7].

In this context, modified Glasgow Prognostic Score (mGPS) and Prognostic Nutrition Index (PNI) and the CALLY Index are not merely nutritional assessment indices but are valuable prognostic tools in predicting the clinical course of patients by considering, in addition to nutrition status, the impact of inflammation on prognosis [8–10].

Incarceration of abdominal hernia, which is capable of giving rise to bowel ischemia and necrosis, is an emergent state in which prompt diagnosis and proper management are key. The incidence of strangulation is one critical factor directly affecting commitment to surgical intervention [11].

Prognostic indicators containing objective measures of inflammatory and immune responses, that is, lymphopenia and significantly raised CRP and declined albumin, could foresee strangulation and requirement of resection in the patient with incarcerated abdominal hernias [12].

## Materials and methods

### Patient selection and study design

This study was approved by the local ethical committee (2024/12). We retrieved the demographic data, laboratory findings, and perioperative details from the hospital database of 125 patients operated for incarcerated abdominal hernia between January 2015 and August 2024. The population was divided into two groups based on the presence or absence of gangrene in the incarcerated bowel loops and omentum. Data collected included age, sex, ASA score, C-reactive protein (CRP) (mg/dL), albumin (mg/dL), lymphocyte count (10^3^/uL), lymphocyte/CRP ratio, Prognostic Nutritional Index (PNI), CRP-Albumin-Lymphocyte Index (CALLY), modified Glasgow Prognostic Score (GPS), omental resection status, mesh usage status, and mortality rates.

Based on the results of the operation, the patients were split into two groups: Patients in Group I did not have resection, while those in Group II had either bowel or omentum resection. The imprisoned intestine is typically aggressively warmed up in our clinic before a final decision regarding its excision is taken after blood flow has been restored. The operative surgeon made the decision on intestinal resection. Thus, Group II was limited to patients involving ischemic bowel and omentum resections. Patients with incarcerated hernias causing obstruction but without intestinal ischemia were included in Group I, along with all other patients without intestinal ischemia. Every pathology report pertaining to intestinal segments or omentum that were removed was regularly documented. Patients over the age of 18 years of age with irreducible abdominal hernias detected by physical examination and those who underwent surgery due to ischemia findings on computed tomography were included in the study. Patients who had reducible abdominal hernias and data that were unavailable were excluded from the study.

## Definitions

$$\begin{aligned}&\mathrm{PNI}=(10\times\mathrm{Serum\; Albumin}\;\lbrack\mathrm g/\mathrm{dL}\rbrack)+(0.005\times\mathrm{Total\; Lymphocyte\; Count}\left[\mathrm{per}\;\mathrm{mm}^3\right])\\ &Cally\mathit=\mathit\lbrack Lymphocyte\; Count\mathit\lbrack cells\mathit/\mu L\mathit\rbrack\mathit\times Albumin\mathit\lbrack g\mathit/dL\mathit\rbrack\mathit)\mathit/CRP\mathit\lbrack mg\mathit/L\mathit\rbrack\\ &\begin{aligned}\mathrm{GPS}=&\mathrm{Score}\;2:\mathrm{CRP}>10\mathrm{mg}/\mathrm{L\; and\; Albumin}<3.5\mathrm g/\mathrm{dL}\\ &\mathrm{Score}\;1:\mathrm{CRP}>10\mathrm{mg}/\mathrm{L\; but\; Albumin}\geq 3.5\mathrm g/\mathrm{dL}\\ &\mathrm{Score}\;0:\mathrm{CRP}\leq 10\mathrm{mg}/\mathrm L\,(\mathrm{regardless\; of\; albumin\; level})\end{aligned}\end{aligned}$$

## Statistical analysis

The distribution of the data was checked for normality using the Kolmogorov–Smirnov test. Descriptive statistics were provided, with continuous variables presented as medians and interquartile ranges (IQR), and categorical variables presented as numbers and percentages. Nonparametric tests, specifically the Mann–Whitney U test, were applied for non-normally distributed data. Chi-square analysis was used for categorical variables. Prospective selective multivariate logistic regression analysis was performed on variables found to be statistically significant. The Hosmer–Lemeshow test was used to evaluate the goodness of fit, with statistical significance set at *p* < 0.05. Data were analyzed using the Statistical Package for the Social Sciences for Windows, version 23 (SPSS Inc., Chicago, IL).

## Results

The study included 66 (52.8%) female patients. The median age of the population was 67 (58–76) years, and 75% had an ASA3 surgical risk. During the operation, 86 (68.8%) patients had necrosis in the omentum, intestine, or both. Among them, 73 (58.4%) patients had only intestinal ischemia, while 23 (18.4%) had only omental necrosis. Postoperative follow-ups revealed mortality in 12 (9.6%) patients (Table 1).

**Table 1:** Demographic data and perioperative findings

Variables		Median(IQR)	Count(%)	
Age, years		67(58–76)		
Gender	Female		66(52.8%)	
	Male		59(47.2%)	
ASA	ASA2		10(8%)	
	ASA3		95(76%)	
	ASA4		20(16%)	
Omental ischemia			23(18.4%)	
Intestinal ischemia			73(58.4%)	
Organ ischemia			86(68.8%)	
Mesh	Presence		87(69.6%)	
Mortality			12(9.6%)	

*IQR* interquartile range, *ASA* American Society of Anesthesiologists

In the group with intestinal and omental ischemia, 52 (60.5%) patients were female, and the median age was 67 (58–78) years. There were statistically significant differences between the groups in terms of gender (*p* = 0.011), CRP (8 [4.5–12.3] vs. 74.95 [25–140]) (*p* < 0.001), albumin (3.98 [3.5–4.3] vs. 3.6 [3–4.02]) (*p* = 0.008), lymphocyte count (1.43 [1.03–2.11] vs. 1.14 [0.73–1.68]) (*p* = 0.024), PNI (48.05 [41–53.65] vs. 40.98 [36–47.75]) (*p* = 0.002), lymphocyte/CRP ratio (0.19 [0.07–0.44] vs. 0.01 [0.006–0.061]) (*p* < 0.001), CALLY < 2.5 (10 [25.6%] vs. 66 [76.7%]) (*p* < 0.001), and GPS 2 (6 [15.4%] vs. 37 [43%]) (*p* < 0.001) (Table 2).

**Table 2:** Univariate regression analysis results of the groups

		no ischemia		organ ischemia		
Variables		Median(IQR)	Count(%)	Median(IQR)	Count(%)	*p*-value
Age, years		66(56–76)		67(58–78)		0.543
Gender	Female		14(35.9%)		52(60.5%)	0.011
	Male		25(64.1%)		34(39.5%)	
ASA	ASA2		4(10.3%)		6(7%)	0.731
	ASA3		28(71.8%)		67(77.9%)	
	ASA4		7(17.9%)		13(15.1%)	
Mesh			36(92.3%)		51(59.3%)	< 0.001
CRP, mg/dL		8(4.5–12.3)		74.95(25–140)		< 0.001
Albumin, mg/dL		3.98(3.5–4.3)		3.6(3–4.02)		0.008
Lymphocyte, 10^3^/uL		1.43(1.03–2.11)		1.14(0.73–1.68)		0.024
PNI		48.05(41–53.65)		40.98(36–47.75)		0.002
Lymphocyte/CRP		0.19(0.07–0.44)		0.01(0.006–0.061)		< 0.001
CALLY	< 2.5		10(25.6%)		66(76.7%)	< 0.001
	≥ 2.5		29(74.4%)		20(23.3%)	
GPS	0		22(56.4%)		7(8.1%)	< 0.001
	1		11(28.2%)		42(48.8%)	
	2		6(15.4%)		37(43%)	
Mortality			1(2.6%)		11(12.8%)	0.072

*IQR* interquartile range, *ASA* American Society of Anesthesiologists, *CRP* C-reactive protein, *PNI* Prognostik nutritional index, *CALLY* CRP-albumin-lymphocyte index, *GPS* Glaskow prognostic score

*p* < 0.05 was considered statistically significant

**Fig. 1:**
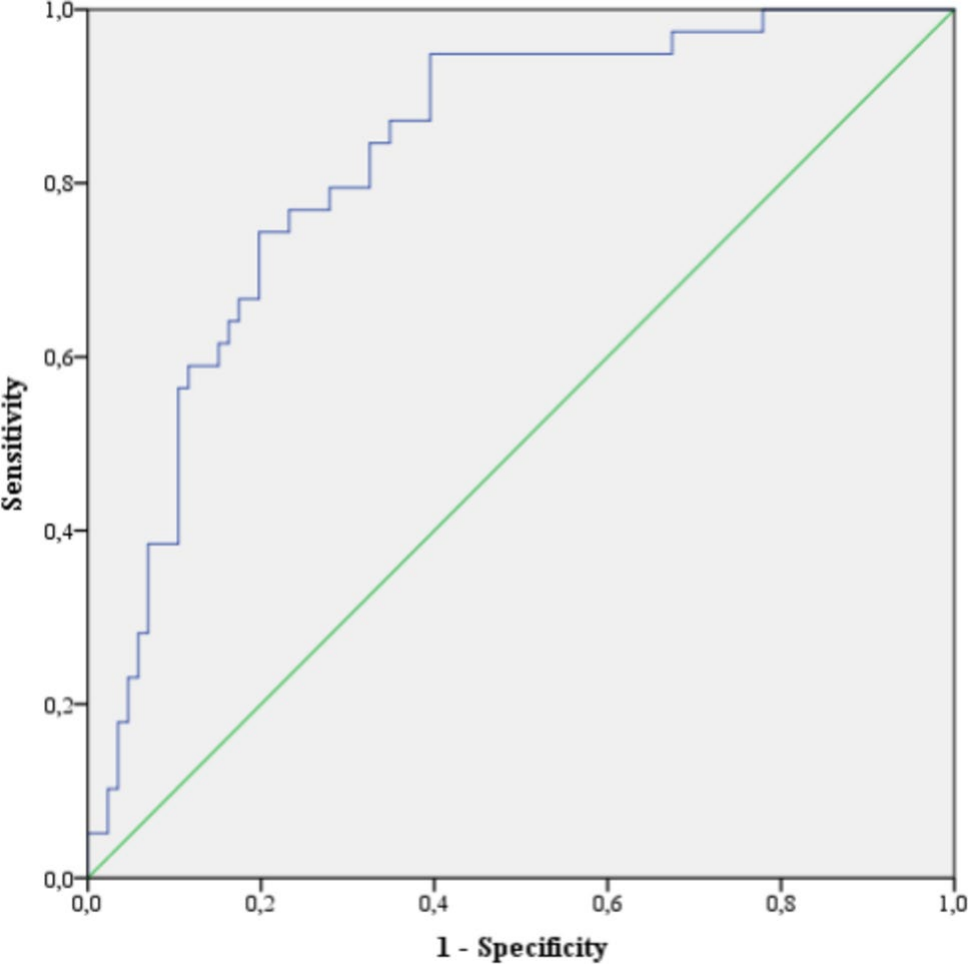
The CALLY value was assessed through ROC analysis, and a cutoff value of < 2.5 was determined (AUC: 0.828 [0.753–0.902])

In the multivariate regression analysis of statistically significant parameters, GPS2 (Odds Ratio: 19.299 [1.77–210.454]; *p* = 0.015) and CALLY < 2.5 (Odds Ratio: 5.397 [1.354–21.513]; *p* = 0.017) were identified as independent risk factors for intestinal ischemia (Table 3). The CALLY value was assessed through ROC analysis, and a cutoff value of < 2.5 was determined (AUC: 0.828 [0.753–0.902]) (Table 4, Fig. 1).

**Table 3:** Multivariate logistic regression analysis

Variables	OR(95% CI)	*p*-value
GPS	1	0.014
GPS1	7.488(1.885–29.754)	0.004
GPS2	19.299(1.77–210.454)	0.015
CALLY < 2.5	5.397(1.354–21.513)	0.017
Albumin, mg/dL	1.926(0.341–10.874)	0.458
Lymphocyte/CRP	1.11(0.45–2.738)	0.821
Gender	1.797(0.69–4.681)	0.23

*OR* odds ratio, *CI* confidence interval, *CALLY* CRP-albuminlymphocyte index, *GPS* Glaskow prognostic score, *CRP* C-reactive protein

*p* < 0.05 was considered statistically significant

**Table 4:** CALLY ROC Analysis Results for Predicting Intestinal Ischemia

		organ ischemia				
		Absence	Presence			
Variable		Count(%)	Count(%)	AUC	95% CI	*p*-value
CALLY	< 2.5	10(25.6%)	66(76.7%)	0.828	0.753–0.902	< 0.001
	≥ 2.5	29(74.4%)	20(23.3%)			

*AUC* area under curve, *CI* confidence interval, *CALLY* CRP-albumin-lymphocyte index

## Discussion

Intestinal ischemia in strangulated abdominal wall hernias is a life-threatening condition that requires an early diagnosis and surgical intervention. Systemic inflammation and nutritional status are related to surgical prognosis; thus, various prognostic indices have been studied for their predictive value in ischemic complications. Among these, albumin-based nutritional indices, such as the CALLY Index, Prognostic Nutritional Index, and modified Glasgow Prognostic Score, have been in wide use in malignancies [8–10] but remain underexplored in acute surgical conditions like incarcerated hernias. Despite the utility of laboratory indices like CALLY and mGPS, instrumental imaging remains essential for definitive diagnosis and surgical planning in cases of suspected intestinal ischemia.

This study is the first to attempt an appraisal of the predictive performance of these indices, particularly the CALLY Index, for intestinal ischemia in strangulated abdominal wall hernias. Our findings indicate that the integration of inflammatory and nutritional markers into risk stratification models may improve clinical decision-making, which could facilitate the early identification of ischemic bowel segments and optimization of surgical outcomes. It should be emphasized that the primary aim of this study was not to detect reversible ischemia but rather to predict cases requiring resection due to irreversible damage, where surgical intervention is mandatory.

The mGPS, is an inflammation- and nutrition based index developed for prognosis in patients with malignancy conditions [13]. This index is calculated using C-reactive protein levels and albumin; such simplicity, rapid assessment, and ease of calculation make this index very valuable in clinical practice. Although a rise in the level of CRP is a manifestation of inflammation, a drop in the level of albumin acts as a negative nutritional indicator in relation to prognosis. The mGPS so far was studied in pancreatic, gastric, colorectal, and gynecological malignancies as a prognostic tool and found to be an effective prognostic marker, but it has not been studied for predicting strangulation and resection in abdominal hernias up to date [14–16]. Our study is the first one to explore the potential of mGPS for the prediction of strangulation and resection in patients with abdominal hernias. A result suggesting that, other than malignancy, mGPS may also have a potential prognostic value in other surgical and inflammatory conditions.

PNI represents a combination measure of lymphocyte count and albumin level for the prediction of postoperative prognosis in cancer patients [9]. This index evaluates two markers: C-reactive protein, an inflammation marker, and albumin, a nutritional marker, and it is commonly applied in several malignancies. Serum albumin is also an accepted negative acute-phase protein, and earlier studies have shown that low serum albumin levels in ischemic stroke patients are associated with increased mortality and prolonged hospitalization [17]. In our series, we wanted to see the predictive value of PNI, for strangulation and resection in abdominal hernias. By evaluating CRP as an inflammation marker and albumin as a negative acute-phase protein, we hypothesized that PNI will be a major factor in the prognosis of strangulation and resection in abdominal hernia patients with incarceration. Our results suggest that PNI is not an independent predictive factor for strangulation and resection, as no significant association was found between PNI levels and the occurrence of these conditions in our analysis.

The CALLY Index is the multiparametric prognostic score of survival that considers inflammation, nutrition, and the immune response in malignancy and different clinical conditions [18]. This index assesses inflammatory response by the level of CRP, the immune function by lymphocyte count, and nutritional status represented by albumin level to provide a comprehensive judgment of the general clinical condition of the patient. Taken together, these three key parameters provide a more holistic view of disease progression by CALLY in a balanced manner. Although, the estimation of its value initially was tried in hepatocellular carcinoma. It was subsequently directed at being an important parameter for anticipating postoperative outcomes in malignancies involving the stomach, esophagus, and the breast [19, 20]. The utilization of albumin, the negative acute phase reactant in CALLY, is as being considered for uses apart from malignancies much like PNI and mGPS, especially regarding inflammation processes. In this respect, the current study has been designed to assess the prognostic value of the CALLY Index for predicting strangulation and resection in incarcerated abdominal hernias. Multivariate analysis indicated that CALLY was an independent prognostic factor for predicting strangulation and resection in patients with abdominal hernias. This again underlines that inflammation, immune response, and nutritional status are important aspects in the clinical management of abdominal hernias. The CALLY Index, due to its reliance on easily accessible laboratory parameters, can serve as a practical tool in preoperative evaluation, especially when rapid risk stratification is needed in emergency settings. Given its simplicity and objectivity, the CALLY Index could potentially be integrated into clinical decision-making algorithms and artificial intelligence tools to assist emergency physicians in identifying high-risk patients needing resection.

The novelty of this study lies in the fact that this will be the first study investigating the possible role of the immuno-nutritional indexs in predicting strangulation and resection in abdominal hernias; thus, it gives an important contribution to show the possible prognostic value of the CALLY Index, mGPS, PNI in surgical and inflammatory conditions other than malignancies.

There are, however, several limitations that need to be considered despite the significant findings from our study. First, there might be a selection bias due to the retrospective nature of the study. Although our findings are promising, they require confirmation through prospective, multicenter studies with larger sample sizes to ensure broader applicability and clinical integration. The sample size, though sufficient for preliminary analysis, is still relatively small. Larger, multicentric, prospective studies should be carried out to validate our findings and ensure generalizability. Moreover, other potential inflammatory and nutritional markers that could affect prognosis were not included in this study, which might give further insight into the complex interplay of inflammation, nutrition, and surgical outcomes.

## Conclusion

This study is the first to investigate the value of the mGPS, PNI and CALLY Index for predicting strangulation and resection in incarcerated abdominal hernias. Of these, mGPS, PNI and CALLY emerged as the independent predictors of resection through multivariate analysis. These observations may suggest that systemic inflammation, immune response, and nutritional status have a crucial role in the prognosis of an incarcerated abdominal hernia and could thus be of assistance in clinical decision-making. However, large-scale prospective studies are needed to present their validation and clinical applicability in routine surgical practice.
